# Up, Down, and All Around: Diagnosis and Treatment of Novel STAT3 Variant

**DOI:** 10.3389/fped.2017.00049

**Published:** 2017-03-13

**Authors:** Michael Alexander Weinreich, Tiphanie P. Vogel, V. Koneti Rao, Joshua D. Milner

**Affiliations:** ^1^Laboratory of Allergic Diseases, National Institute of Allergy and Infectious Diseases (NIAID), National Institutes of Health (NIH), Bethesda, MD, USA; ^2^Center for Human Immunobiology, Texas Children’s Hospital, Baylor College of Medicine, Houston, TX, USA; ^3^Division of Intramural Research, Laboratory of Clinical Infectious Diseases, National Institute of Allergy and Infectious Diseases (NIAID), National Institutes of Health (NIH), Bethesda, MD, USA

**Keywords:** STAT3 transcription factor, gain-of-function mutations, autoimmune lymphoproliferative syndrome, monogenic autoimmunity, human immunology

## Abstract

The number of identified monogenic causes of childhood-onset autoimmunity due to nodal and extranodal lymphoproliferation has increased. These pathogenic genetic variants provide the potential for pathway-specific treatment. Novel variants also require pathway-specific verification. In this report, we describe a 14-year-old patient with a novel variant in STAT3. We report clinical and laboratory findings that support STAT3 p.G419R as a novel pathogenic STAT3 gain-of-function variant.

## Introduction

In recent years, multiple monogenic causes of early-onset autoimmunity/lymphoproliferation have been identified. These include *CTLA4, LRBA, PIK3CD, PIK3R1*, and STAT3 gain-of-function (GOF) (1–9). CTLA4 and LRBA patients can present with autoimmune cytopenias, lymphoproliferation, hypogammaglobulinemia, and lymphocytic infiltration of non-lymphoid organs. Patients with *PIK3CD* and *PIK3R1* variants leading to increased signaling can present with lymphoproliferation, recurrent sinopulmonary infections, progressive airway damage, and chronic herpesvirus viremia. Patients with STAT3 GOF can present with cytopenias, lymphoproliferation, multi-organ autoimmunity, short stature, and hypogammaglobulinemia. These can be added to previously known immune dysregulatory disorders, such as immunodysregulation polyendocrinopathy enteropathy X-linked syndrome (IPEX), autoimmune polyendocrinopathy, candidiasis ectodermal dystrophy/dysplasia, and autoimmune lymphoproliferative syndrome (ALPS) due to variants in FOXP3, AIRE or FAS, FAS ligand, and caspase-10 (10–12), respectively. The identification of new syndromes makes it more fruitful to search for pathogenic variants in symptomatic patients and can potentially provide pathway-specific management options.

## Case Report

The patient is a 14-year-old Caucasian male born full term with no complications. He developed lymphadenopathy at age 3 leading to an excisional biopsy of an axillary node, which showed normal lymph node tissue. His mother denied noticeable persistent lymphadenopathy. At age 9, he developed autoimmune anemia and thrombocytopenia responsive to IVIg and steroids. Lymphadenopathy was not documented at this time. This led to a work up for autoimmune lymphoproliferative syndrome (ALPS). He was found to have normal numbers of double negative alpha/beta T cells with no B220+ DNT cells. He had two subsequent episodes of autoimmune hemolytic anemia. The most recent occurred at age 11 and he was treated with a 4-week course of rituximab. Between episodes, he was asymptomatic and was taking no medications. The patient had no history of significant or severe infections.

At age 13, the patient developed back pain, fever, shortness of breath, and cough. He did not improve despite multiple rounds of antibiotics for presumed pneumonia. He was admitted for IV antibiotic treatment. A chest CT showed mediastinal adenopathy and irregular nodular pulmonary infiltrates. A sputum culture was positive for *Candida* and the patient was treated with antifungals. In total, he received 29 days of amphotericin and micofungin. A bronchoscopy culture was negative for bacteria and fungus. A lung biopsy showed an atypical interstitiital lymphoplasmacytic infiltrate, suggesting an underlying lymphoproliferative process. Bacterial and fungal cultures of the lung were also negative. During his admission he was noted to have IgG < 155 mg/dL (low), IgM 354 mg/dL (high), and IgA 8.7 mg/dL (low). His hypogammaglobinemia was treated with IVIg. He gradually improved and was discharged.

**Figure 1:**
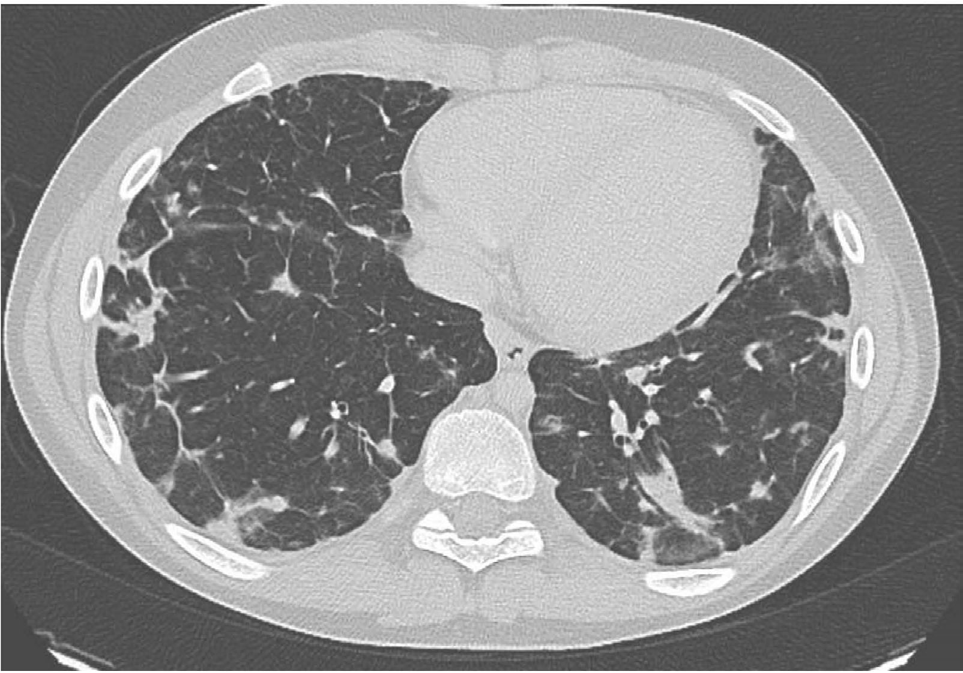
**Chest CT of patient prior to treatment with mycophenolic acid showing multiple infiltrates**.

He was then referred to our institution for further work up and treatment. Written and informed consent was obtained from the patient and his family members. This study was approved by the Institutional Review Board of the National Institutes of Health. At presentation he reported being in his normal state of health. He was greater than 50th percentile for height and weight. He has no history of arthritis. He reported shortness of breath while playing baseball. His oxygen saturation was 100% at rest but decreased to 92% during a 6-minute walk test. His CT scan showed multiple nodules throughout the lung (Figure 1). Of note, many larger lesions seen in prior CT scans had decreased in size or resolved and lesions in new locations were appreciated. Lymphadenopathy and splenomegaly were also noted.

**Figure 2:**
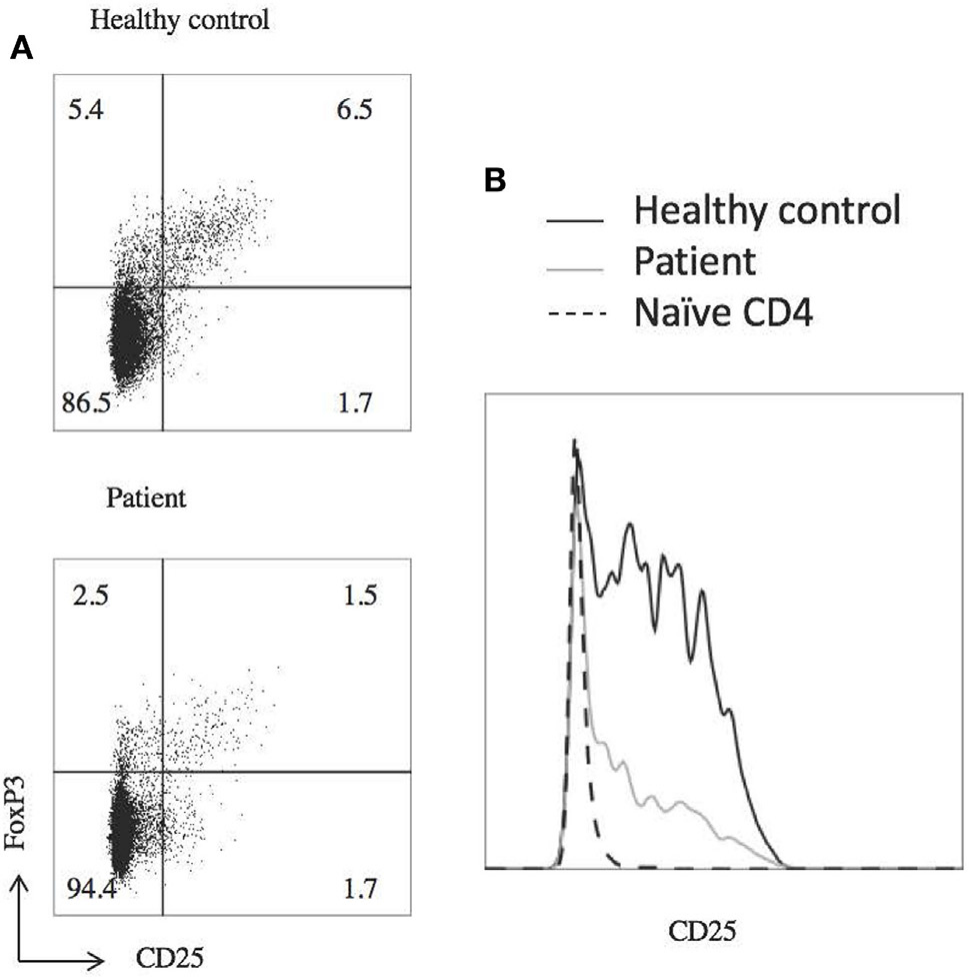
**Patient has decreased T regulatory cell (T reg) numbers and expresses less CD25**. **(A)** Flow cytometric analysis of T reg populations, gated on CD4+ T cells. **(B)** T regs have decreased CD25 expression, gated on CD4+ FOXP3+ CD127low cells. Dashed line shows naïve (CD45RO negative) T cells from a healthy control to show negative CD25 staining. Experiments were repeated at least twice with at least two healthy controls for each experiment.

**Figure 3:**
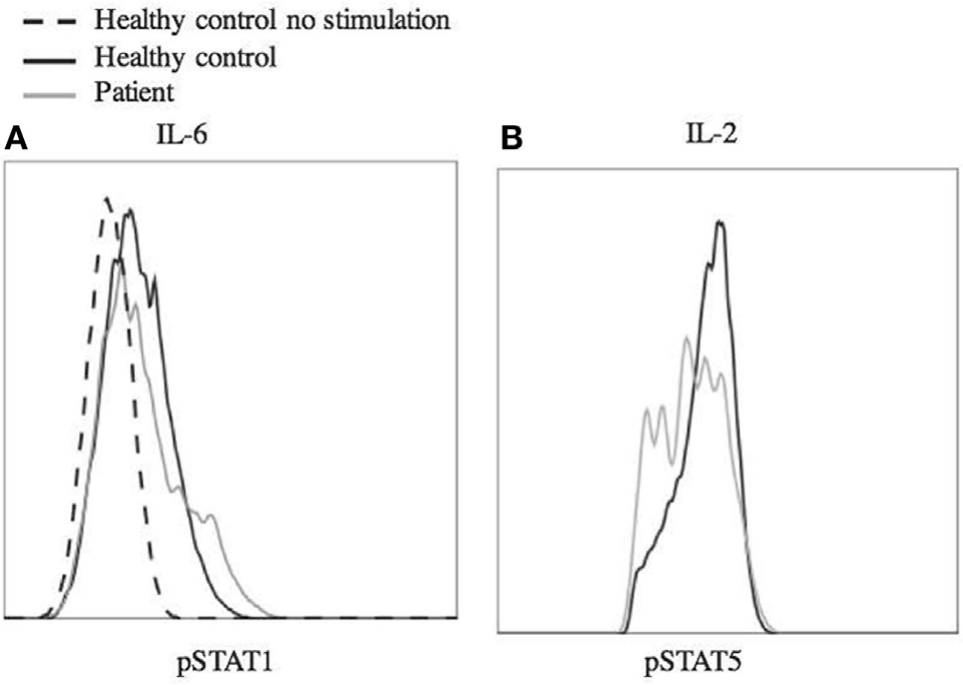
**Phosphorylation of STAT1 and STAT5 in response to stimulus**. Peripheral blood mononuclear cells from the patient and a healthy control were stimulated with IL-6 (25 ng/mL) **(A)** and IL-2 (200 ng/mL) **(B)** for 20 min. The cells were fixed using 4% paraformaldehyde and permeabilized with methanol, then stained with antibody and analyzed by flow cytometry. Cells were gated on CD4+ **(A)** or CD4+, pSTAT5+ **(B)** cells. Dashed line shows unstimulated cells from a healthy control. Experiments were repeated at least twice with at least two healthy controls for each experiment.

Laboratory studies showed WBC of 10.36 K/dL, hemoglobin 12.6 g/dL, and platelet count of 386 K/μL. CMV and EBV were not detected by PCR of the blood. The patient did have abnormalities with decreased IgA (5 mg/dL) and IgG (135 mg/dL), elevated IgM (484 mg/dL), and IgE level was <1.0 IU/mL. Analysis of B cells showed normal total (13.4%, 256/μL) and switched memory (CD20+, CD27+, IgM−, 0.3%, 6/μL) B cells, and undetectable unswitched memory B cells (CD20+, CD27+, IgM+). B12 level was 921. At the time of this analysis, he had last received IVIg 3 months prior and rituximab treatment was more than 2 years prior. Laboratory analysis further revealed decreased regulatory T cells with decreased CD25 expression (Figure 2). There were variable responses of phosphorylation of STAT (pSTAT) proteins to cytokine stimuli, which did not lead to a clear pattern (Figure 3) (8).

**Figure 4:**
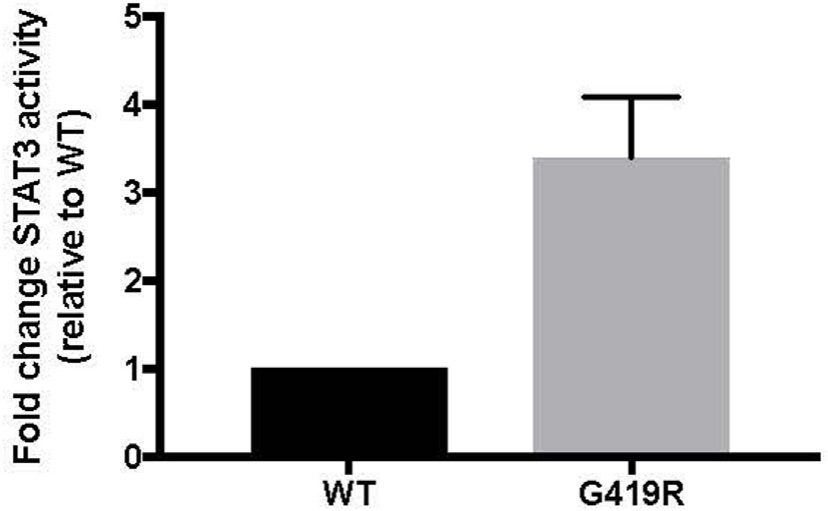
**p.G419R STAT3 variant shows increased STAT3 activity at baseline**. Wild-type (WT) or mutant STAT3 plasmids were co-transfected into STAT3-deficient cells along with a STAT3-responsive luciferase reporter, followed by measurement of luciferase at 48 h (8). Averages shown are from four independent experiments.

Targeted genetic testing using a panel of more than 150 known and predicted immunity-related genes, including *CTLA4, LRBA, STAT3, PIK3CD, PIK3R1, FOXP3, AIRE, FAS, FASLG*, and *CASP10*, yielded only one rare variant of interest. A novel, heterozygous missense mutation in *STAT3* c.1255G>C, p.G419R was found in the DNA binding domain just a few amino acids away from several other reported STAT3 GOF mutations and confirmed by Sanger sequencing. This variant was predicted to be deleterious by modeling with CADD phred score of 25.3 (13). Inheritance is unknown. GOF activity was confirmed using a luciferase assay, as previously reported (Figure 4) (8).

## Discussion

Autoimmune lymphoproliferative syndrome-FAS is the best known of the autoimmune/lymphoproliferative disorders. In a case such as this patient when the number DNT cells, elevated in ALPS, are normal or there is lymphoproliferative infiltration of non-lymphoid organs, such as the lung, further testing for an alternative genetic diagnosis should be considered. Both loss-of-function (LOF) and GOF pathogenic variants have been described in STAT3-related disease. STAT3 LOF, which leads to autosomal dominant hyper-IgE syndrome does not fit this patient’s phenotype (14). However, his presentation is similar to patients with STAT3 GOF which can mimic ALPS, IPEX or IPEX-like disorders, and STAT5b-deficiency (8, 9, 15).

It is not clear that the *Candida* found during this patient’s hospitalization was pathogenic and he does not otherwise have a history of infections. A subset of STAT3 GOF patients can have infections expected by hypogammaglobulinemia and mycobacterial infection has been reported in one patient. We do not have IgG levels prior to rituximab treatment. While prolonged hypogammaglobulinemia post-rituximab is possible, the majority of patients treated for autoimmunity do not require Ig replacement 2 years after rituximab treatment (16, 17). Thus, his hypogammaglobulinemia is likely due to intrinsic immune dysregulation as reported with STAT3 GOF.

Given the entire clinical picture, his pulmonary pathology is most consistent with lymphoproliferation, a finding in multiple STAT3 GOF patients. Despite reporting only mild exercise intolerance on presentation to our institution, the patient was found to have significant pulmonary infiltration on CT scan. His lung biopsy was consistent with a lymphoproliferative process. In a retrospective study of patients with ALPS-like phenotype and findings on pulmonary CT scan, 89% were asymptomatic (18). Because of the patient’s exercise intolerance and extensive pulmonary lesions he was started on immunosuppression. In addition to IVIg to treat his hypogammaglobulinemia, mycophenolic acid was initiated for his lymphoproliferative lung disease, which he has tolerated well. The patient does not have bronchiectasis, and his pulmonary changes may be reversible. Repeat chest imaging is planned. Mycophenolic acid has been used successfully in similar lymphoproliferative patients, including some that were later found to have STAT3 GOF (19). The importance of the diagnosis and confirmation of this STAT3 GOF variant as pathogenic arises if this patient requires additional treatment as potential treatments for STAT3 GOF patients include upstream inhibitors, such as tocilizumab (anti-IL-6 receptor monoclonal antibody) and Janus kinase inhibitors. Specific STAT3 inhibitors are currently being evaluated in clinical trials for cancer.

As genetic sequencing becomes more prevalent, the importance of verification of genetic variants will become more important. In this case, the clinical presentation and the T regulatory cell profile were consistent with that of other known STAT3 GOF patients. However, interpretation of laboratory studies may be complicated in STAT3 GOF patients by systemic immune suppressants often administered prior to diagnosis. In this case, the patient did not have multi-organ autoimmunity or short stature and the pSTAT responses to multiple cytokines did not yield a clear picture of pSTAT1 and pSTAT5 suppression as seen in primary cells from a number of STAT3 GOF patients (8). Therefore, we functionally confirmed that the STAT3 p.G419R variant leads to a 3.7-fold increase of STAT3 transcriptional activity at baseline compared to wild-type STAT3 (Figure 4).

We report clinical and laboratory findings that support STAT3 p.G419R as a novel pathogenic STAT3 GOF variant. This case illustrates the importance of a genetic diagnosis in syndromes of lymphoproliferation and immunedysregulation. Proper diagnosis can lead to decreased use of unnecessary antimicrobials, more expedient use of immune suppression and appropriate monitoring.

## Ethics Statement

This study was carried out in accordance with the recommendations of NIH IRB with written informed consent from all subjects. All subjects gave written informed consent in accordance with the Declaration of Helsinki. The protocol 14-I-0206 was approved by the NIH IRB.

## Author Contributions

Conception or design of the work: MW and JM. Data collection: TV and MW. Data analysis and interpretation: MW, TV, and JM. Drafting the article: MW. Critical revision of the article: TV, VR, and JM. Final approval of the version to be published and agreement to be accountable for all aspects of the work in ensuring that questions related to the accuracy or integrity of any part of the work are appropriately investigated and resolved: MW, TV, VR, and JM.

## Conflict of Interest Statement

The authors declare that the research was conducted in the absence of any commercial or financial relationships that could be construed as a potential conflict of interest.
